# A self-supervised classification model for endometrial diseases

**DOI:** 10.1007/s00432-023-05467-7

**Published:** 2023-11-10

**Authors:** Yun Fang, Yanmin Wei, Xiaoying Liu, Liufeng Qin, Yunxia Gao, Zhengjun Yu, Xia Xu, Guofen Cha, Xuehua Zhu, Xue Wang, Lijuan Xu, Lulu Cao, Xiangrui Chen, Haixia Jiang, Chaozhen Zhang, Yuwang Zhou, Jinqi Zhu

**Affiliations:** 1 Quzhou People’s Hospital, The Quzhou Affiliated Hospital of Wenzhou Medical University, Quzhou, 324000 Zhejiang China; 2 Tianjin Normal University, Tianjin, 300387 China; 3 The Second People’s Hospital of Quzhou, Quzhou, 324000 Zhejiang China; 4 Kaihua County People’s Hospital, Quzhou, 324300 Zhejiang China; 5 Changshan County People’s Hospital, Quzhou, 324200 Zhejiang China; 6 People’s Hospital of Quzhou Kecheng, Quzhou, 324000 Zhejiang China; 7 Quzhou Maternal and Child Health Care Hospital, Quzhou, 324000 Zhejiang China

**Keywords:** Endometrial cancer, Transvaginal ultrasound, Convolutional neural network, Self-supervised learning

## Abstract

**Purpose:**

Ultrasound imaging is the preferred method for the early diagnosis of endometrial diseases because of its non-invasive nature, low cost, and real-time imaging features. However, the accurate evaluation of ultrasound images relies heavily on the experience of radiologist. Therefore, a stable and objective computer-aided diagnostic model is crucial to assist radiologists in diagnosing endometrial lesions.

**Methods:**

Transvaginal ultrasound images were collected from multiple hospitals in Quzhou city, Zhejiang province. The dataset comprised 1875 images from 734 patients, including cases of endometrial polyps, hyperplasia, and cancer. Here, we proposed a based self-supervised endometrial disease classification model (BSEM) that learns a joint unified task (raw and self-supervised tasks) and applies self-distillation techniques and ensemble strategies to aid doctors in diagnosing endometrial diseases.

**Results:**

The performance of BSEM was evaluated using fivefold cross-validation. The experimental results indicated that the BSEM model achieved satisfactory performance across indicators, with scores of 75.1%, 87.3%, 76.5%, 73.4%, and 74.1% for accuracy, area under the curve, precision, recall, and F1 score, respectively. Furthermore, compared to the baseline models ResNet, DenseNet, VGGNet, ConvNeXt, VIT, and CMT, the BSEM model enhanced accuracy, area under the curve, precision, recall, and F1 score in 3.3–7.9%, 3.2–7.3%, 3.9–8.5%, 3.1–8.5%, and 3.3–9.0%, respectively.

**Conclusion:**

The BSEM model is an auxiliary diagnostic tool for the early detection of endometrial diseases revealed by ultrasound and helps radiologists to be accurate and efficient while screening for precancerous endometrial lesions.

## Introduction

Endometrial cancer is the sixth most commonly diagnosed cancer in women (Sung et al. 2021) and encompasses a group of malignant epithelial tumours that develop in the endometrium (Colombo et al. 2016). It mainly affects women with postmenopausal, particularly those with a history of obesity, hypertension, and familial cancer, and, recently, has become increasingly common (Passarello et al. 2019). Endometrial lesions include endometrial polyps, hyperplasia, and cancer, with the latter being the most severe (Valentin 2014). The 5 years survival rate for patients in stage I endometrial cancer can reach 80–90%, whereas those in stages III or IV have significantly lower survival rates, of 50–65% and 15–17% (Makker et al. 2021), respectively. Early diagnosis plays a pivotal role in effective treatment of endometrial cancer.

Histopathological examination of the endometrium is considered the gold standard for diagnosing endometrial lesions in clinical practice (Karaca et al. 2022). Endometrial tissues can be obtained using diagnostic curettage or hysteroscopic dilatation and curettage (Vitale et al. 2023). However, the high cost and associated risk of complications (Dijkhuizen et al. 2003; Williams and Gaddey 2020) make histopathological examination a less favourable choice for early diagnosis (Wong et al. 2016). In contrast, ultrasound imaging, specifically transvaginal ultrasound (TVU), is a safe, well-tolerated (Salman et al. 2016), non-invasive, low-cost, and affordable method that can identify endometrial abnormalities such as thickening and atypical imaging features (e.g., cystic endometrium, intraluminal fluid, and suspected polyps), serving as the basis for diagnosing endometrial diseases (Aggarwal et al. 2021). Therefore, ultrasound imaging is the method of choice for the early diagnosis of endometrial diseases. However, there is considerable variability in the evaluation results among different radiologists when assessing the same ultrasound image, mainly because of the subjective nature of ultrasound-based pathological evaluation, which relies heavily on the experience of radiologists. Developing a stable and objective computer-aided diagnosis model can effectively reduce the subjectivity associated with the diagnoses of radiologists.

Deep learning is reported as a promising tool for the classification of endometrial diseases (Zhang et al. 2022, 2021; Li et al. 2022; Urushibara et al. 2022; Mao et al. 2022; Tao et al. 2022; Zhao et al. 2022; Sun et al. 2020). However, the studies on this topic have several issues: (1) incomplete classification, such as distinguishing between endometrial and non-endometrial cancers; (2) lack of sample diversity, as samples are often obtained from the same hospital or imaging device; and (3) existing studies primarily rely on magnetic resonance imaging (MRI) and histopathological images (HI) for classification, but these methods have drawbacks (Szkodziak et al. 2014; 2017) such as high cost, time-consuming procedures, dependence on expert interpretation, potential complications from invasive techniques, and limited access to MRI equipment in certain healthcare facilities. In contrast, ultrasound imaging offers a non-invasive and cost-effective approach, provides real-time imaging, and is widely accessible. Consequently, ultrasound imaging has emerged as the preferred method for early detection of endometrial diseases.

Our study focused on three prevalent endometrial diseases using TVU images: endometrial polyps, hyperplasia, and cancer. This paper proposes a joint training approach that integrates an original task with a self-supervised task. Specifically, we performed auxiliary training on the original task by utilizing self-supervised images generated through the original image rotation. The predictions from all the images, including the original and rotated images, were then aggregated to improve the overall prediction accuracy. Furthermore, self-distillation techniques and a voting ensemble strategy were performed to reduce the variance and enhance the generalisation and robustness of the model. This study aimed to establish an auxiliary diagnostic tool for endometrial disease classification by leveraging both original and self-supervised labels. This approach effectively addressed the challenges of limited sample diversity and incomplete classification in the field of endometrial disease classification, thereby overcoming the issues related to the low generalisation and robustness of the model.

## Methods

### Dataset

**Fig. 1:**
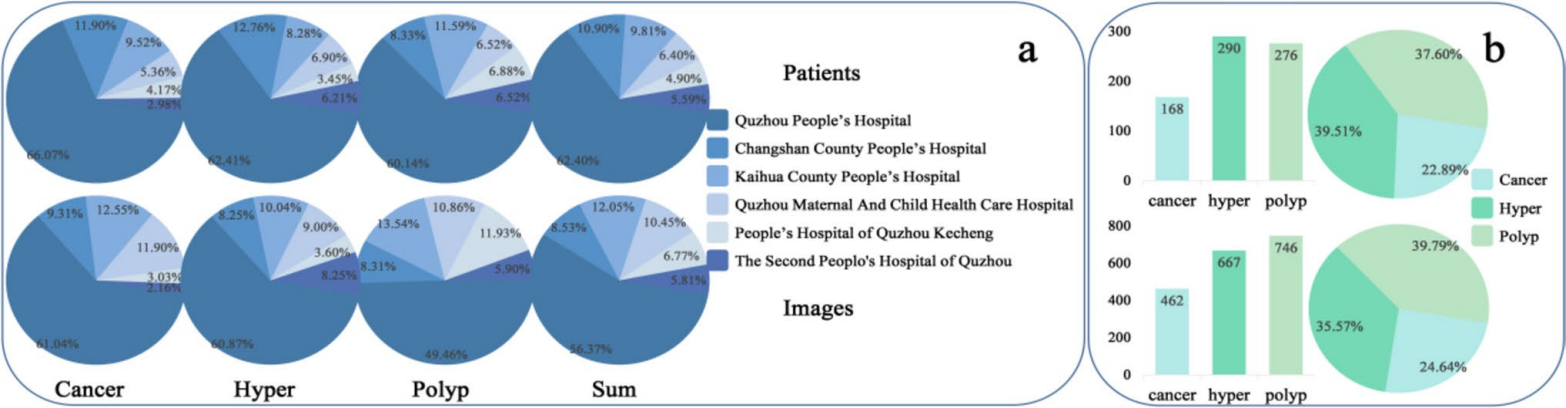
Statistical distribution of the dataset, the upper part is patients and the lower part is images: **a** Distribution ratios of the number of patients and number of images with endometrial polyps, hyperplasia and cancer across different hospitals. **b** The total number and distribution ratio of the number of patients and the number of images of three endometrial diseases

Ethics committee approval was granted by the local institutional ethics review board and the requirement for informed consent was waived for this retrospective study. This study collected ultrasound images from patients aged 40–70 years who underwent TVU in Quzhou City, Zhejiang Province, including Quzhou People’s Hospital, Quzhou Maternal And Child Health Care Hospital, The Second People’s Hospital of Quzhou, People’s Hospital of Quzhou Kecheng, Changshan County People’s Hospital, and Kaihua County People’s Hospital between January 2018 and March 2023. Ultrasound images were obtained from different models of machines, including Samsung WS80A, GE Voluson E10, PHiliPsQ5, Mindray Resona 6s, GE Voluson E8, and PHiliPsQ7, which have several advantages, including data diversity and richness and cross-device validation, thereby enhancing the generalisation of the model. All images were initially stored in the DICOM format and subsequently converted to the JPG format. During the image collection process, the following exclusion criteria were applied: (1) images of patients with multiple uterine disorders, and (2) images with noticeable defects or blurring. These exclusion criteria were implemented to ensure the inclusion of high-quality images and provide reliable training data for the study. A total of 1875 images from 734 patients were obtained, including 462 images from 168 patients with endometrial cancer, 667 images from 290 patients with endometrial hyperplasia, and 746 images from 276 patients with endometrial polyps. The distributions of the cases and images are summarised in Fig. 1.

### Data processing

First, the removal of hospital and patient information from the image ensured that the image information related to the endometrium was fully preserved, which effectively reduced the impact of irrelevant information on the performance of the model. Second, data augmentation techniques, such as random horizontal flipping, were applied to the training set, which enhanced the generalisation and robustness of the model. Finally, all the images were resized to a uniform size of 224 × 224 pixels and normalised, which accelerated the convergence of the model.

### Statistical analysis

The classification results were evaluated using fivefold cross-validation (Wong and Yeh 2019). First, the dataset was randomly partitioned into five subsamples of equal size. Each subsample was then sequentially used as the test set, and the remaining subsamples served as the training set. The accuracy of each test was recorded, and the average accuracy across all tests was calculated to estimate the performance of the algorithm.

To evaluate the performance of the classification of the model, we used a receiver operating characteristic (ROC) curve and a confusion matrix. The confusion matrix included the number of true positive (TP), false positive (FP), false negative (FN), and true negative (TN) classifications. By comparing the predicted results with the actual labels, the confusion matrix enabled the evaluation of the accuracy of the model across different categories. In addition, we utilised metrics such as accuracy, area under the curve (AUC), precision, recall, and F1 score to further evaluate the performance of the model. By conducting a comprehensive analysis of the ROC curve, confusion matrix, and the aforementioned evaluation metrics, we gained a more comprehensive understanding of the performance of the classification of the model.

In the evaluation, Python 3.7.0 was utilized to calculate various statistics and metrics, such as accuracy and recall. Matplotlib was used for visualisation operations such as drawing ROC curves, confusion matrices, and other graphical representations, which enabled a more intuitive observation and analysis of the performance of the model.

### Model

**Fig. 2:**
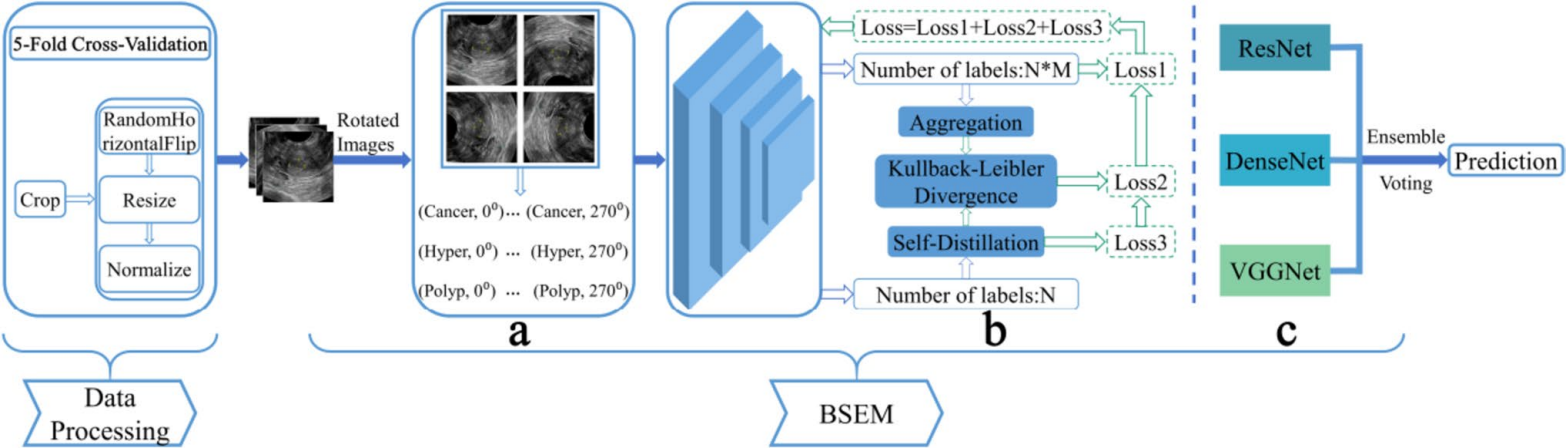
Method overview: **a** Label augmentation; **b** Self-distillation; **c** Voting ensemble

This study investigated the issues of limited sample diversity and incomplete classification in endometrial disease classification, and proposed an approach to learning the joint distribution of raw labels and self-supervised labels by utilizing TVU images, as displayed in Fig. 2. We used a label augmentation (Lee et al. 2020; Xie et al. 2021; Xie et al. 2023), as depicted in Fig. 2a. Specifically, in the dataset, the original labels were *N* = 3, and the labels obtained from self-supervised rotation were *M* = 4. By learning the joint probability distribution of all possible combinations, we obtained a total of N*M = 12 labels. Each transformation was assigned a distinct self-supervised label, enabling aggregation of all transformations for prediction. To improve the generalization of the model, we utilized the self-distillation technique (Hinton et al. 2015), as illustrated in Fig. 2b. Lastly, to enhance the robustness of the model while reducing variance, we used a voting ensemble strategy (Rojarath et al. 2016; Liu et al. 2022), as shown in Fig. 2c.

The ultrasound images were used as input; the label for the original images was *y* = {0, 1, 2}, and the self-supervised label *M*_*j*_ = {0°, 90°, 180°, 270°} represented the image rotation operations. Self-supervised learning is advantageous by training the model to predict transformed data, which compels the model to learn the underlying structure and features within the data, leading to enhanced performance and generalisation of the model. To leverage the benefits of self-supervised learning to the fullest extent possible, we used a joint training strategy that combined both original labels and self-supervised labels. Consequently, the training objective could be expressed as:

1 $$L_{JC} = L_{CE} \left( {f\left( {y,j} \right)} \right),$$

where *L*_*ce*_ represents the cross-entropy loss function. Because each transformation was assigned a different self-supervised label, all transformations were aggregated to improve the performance of the model, where the aggregated probability was denoted as *P*_*agg*_. We introduced a self-distillation operation, which distilled the aggregated knowledge *P*_*agg*_ to another classifier *σ*. We then used the Kullback–Leibler divergence (Kim et al. 2021) to measure the similarity between aggregated and self-distilled classifiers, optimising the performance of the model. Hence, the following objectives were optimised:

2 $$L_{BSEM} = L_{JC} + L_{KL} + L_{SD} = L_{CE} \left( {f\left( {y,j} \right)} \right) + D_{KL} \left( {P_{agg} \left\| \sigma \right.} \right) + L_{CE} \left( {\sigma \left( y \right)} \right).$$

Optimising the aforementioned objectives allowed the model to learn the underlying structure and characteristics of the data through self-supervised tasks, leading to enhanced performance and generalisation of the model. To enhance the robustness and generalisation of the model further, we used a voting ensemble learning strategy. This strategy involves integrating multiple models and making decisions based on the principle of majority rule, thereby reducing the model variance and improving the overall performance. In our approach, we utilised DenseNet, VGGNet, and ResNet as backbone architectures for feature extraction, and combined them using voting strategies.

During the training process, we used a pre-training strategy and imported the pre-training parameters into the model. The Adam optimisation algorithm was utilised with a batch size of 64, weight decay of 1e–4, and a learning rate of 0.01. The training was conducted for 150 iterations.

## Results

The based self-supervised endometrial disease classification model (BSEM) was evaluated using several performance metrics: accuracy, AUC, precision, recall, and F1 score. The results obtained for these metrics were 75.1%, 87.3%, 76.5%, 73.4%, and 74.1%, respectively, which provided a comprehensive assessment of the classification performance and properties of the model.

**Fig. 3:**
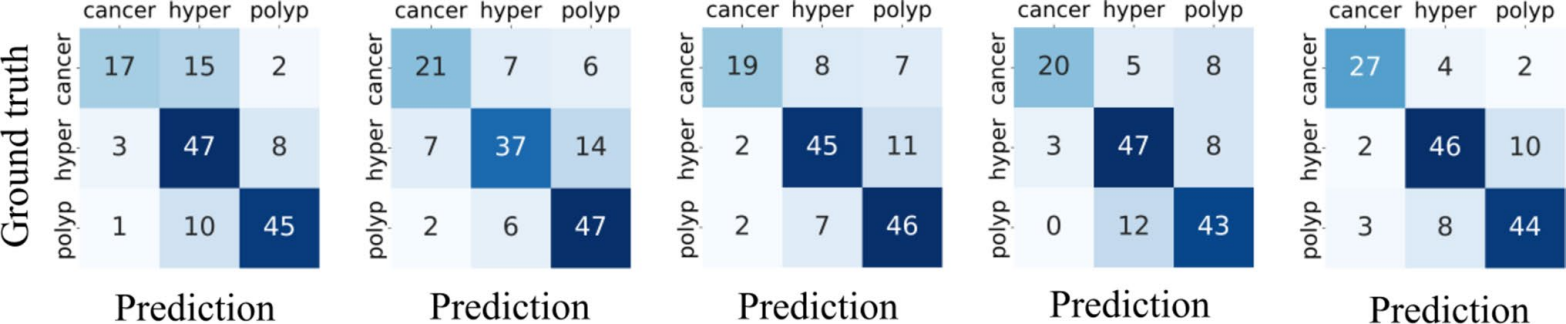
Depiction of the confusion matrix results obtained from the fivefold cross-validation, in which the vertical axis represents the true labels, and the horizontal axis represents the predicted results

**Fig. 4:**
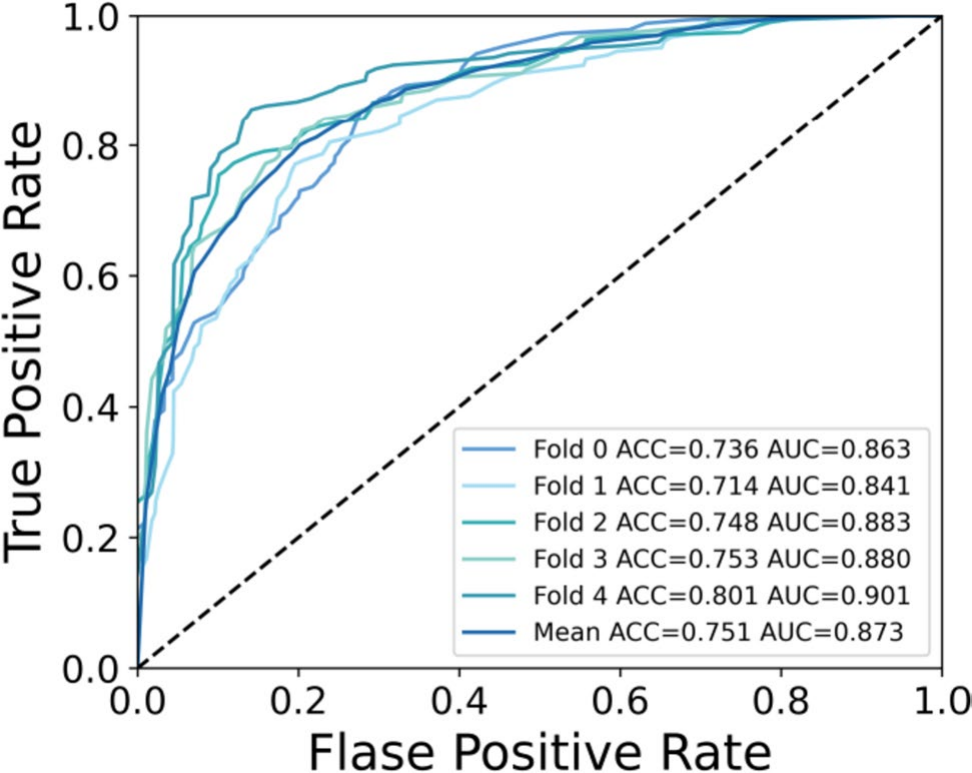
ROC curves for each fold in the fivefold cross-validation, along with the average ROC curve

To gain a deeper understanding of the BSEM classification results across different datasets, we generated separate confusion matrices for each fold, as shown in Fig. 3. Furthermore, to visually illustrate the true positive rate and false positive rate of the model at different thresholds, we generated a ROC curve. The ROC curve results for the proposed model are shown in Fig. 4.

**Fig. 5:**
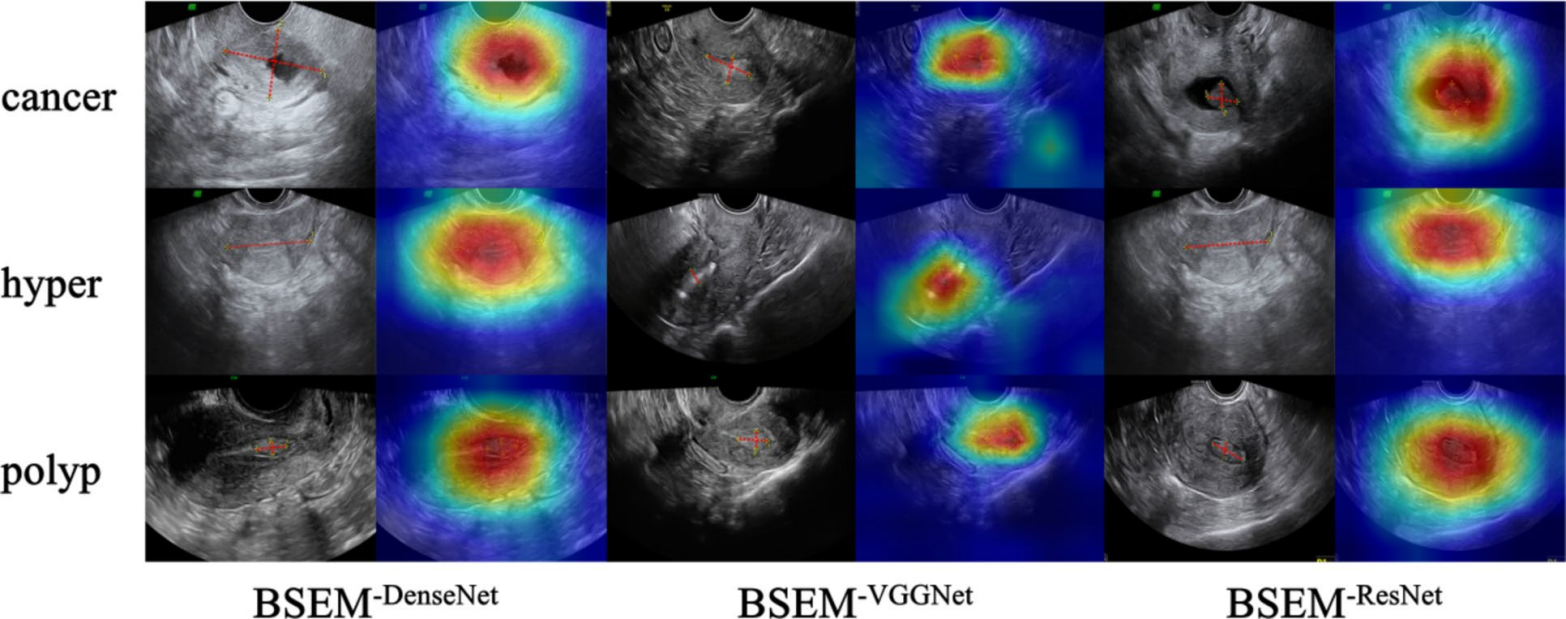
CAM-based visualisation of individual models before the voting ensemble strategy. The grayscale image represents the original input, and the red dotted line denotes the lesion area. The colour image corresponds to the CAM output, with the red area indicating a considerable contribution to the classification results

To visualise the classification effect of our model, we utilised Class Activation Mapping (CAM) technology, which highlights regions that significantly contribute to the classification results. In Fig. 5, the grayscale image represents the original input and the colour image represents the CAM-processed output.

### Comparison with the baseline

Table 1 presents the performance enhancements achieved by the BSEM model for accuracy, AUC, precision, recall, and F1 score when compared to the baseline models. Compared to the ResNet, DenseNet, VGGNet, ConvNeXt, VIT, and CMT baseline models, the BSEM model achieved increased performances of 4.4%, 3.9%, 3.9%, 4.8%, and 4.8%; 3.3%, 3.2%, 4.2%, 3.1%, and 3.3%; 5.1%, 5.5%, 5.0%, 5.7%, and 5.7%; 5.2%, 5.1%, 7.2%, 5.0%, and 5.6%; 6.6%, 7.0%, 7.4%, 6.7%, and 6.8%; and 7.9%, 7.3%, 8.5%, 8.5%, and 9.0%, respectively, for accuracy, AUC, precision, recall, and F1 score, respectively.

**Table 1:** Performance metrics of the BSEM and baseline models

Method	Accuracy	AUC	Precision	Recall	F1 score
ResNet	70.7%	83.4%	72.6%	68.6%	69.3%
DenseNet	71.8%	84.1%	72.3%	70.3%	70.8%
VGGNet	70.0%	81.8%	71.5%	67.7%	68.4%
ConvNeXt	69.9%	82.2%	69.3%	68.4%	68.5%
VIT	68.5%	80.3%	69.1%	66.7%	67.3%
CMT	67.2%	80.0%	68.0%	64.9%	65.1%
BSEM	75.1%	87.3%	76.5%	73.4%	74.1%

**Fig. 6:**
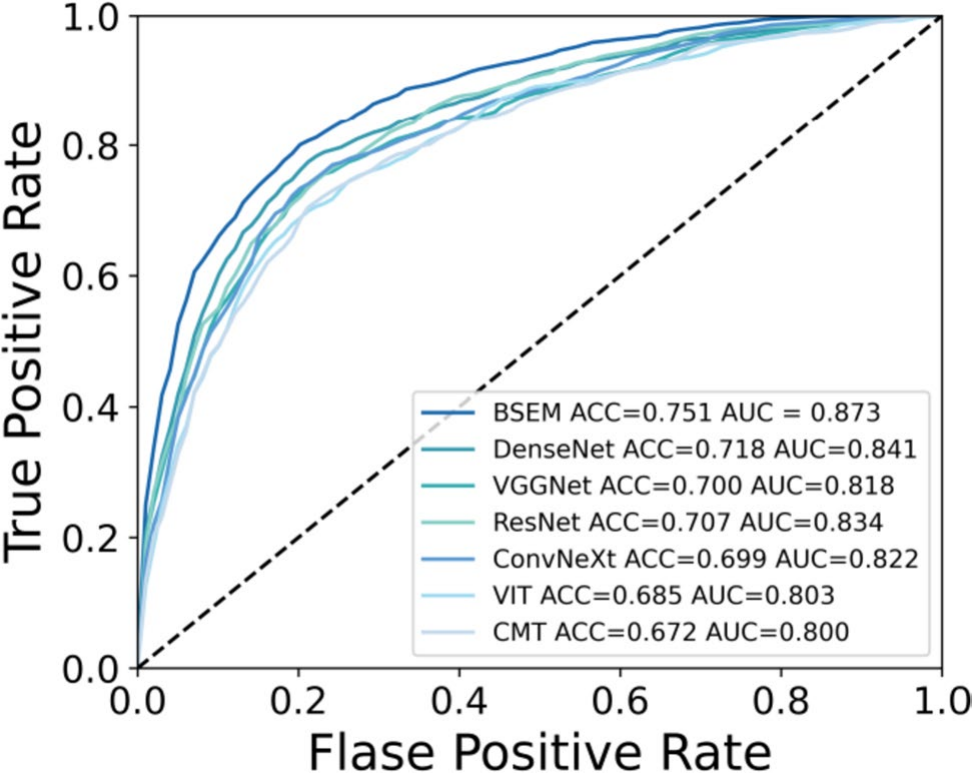
ROC curves of the BSEM and baseline models

The ROC curve is a crucial performance metric for evaluating classification models. Figure 6 provides a visual comparison between the proposed model and the baseline model, showing their performances on the ROC curve. The figure illustrates that, across various thresholds, the BSEM model exhibited a higher true-positive rate and a lower false-positive rate than the baseline model. This observation indicated the superior classification ability and robustness of the proposed model.

### Ablation experiments

To evaluate the effectiveness of each component, we performed ablation studies by adding the following models: BSEM^−ResNet^, BSEM^−DenseNet^, and BSEM^−VGGNet^, representing the models without a voting ensemble strategy, and BSEM^−SD^, representing the model without a self-distillation operation.

Our findings demonstrated that the proposed BSEM model exhibited substantial improvements in various performance metrics when compared with the model without the voting ensemble strategy. Specifically, when compared with the BSEM^−ResNet^, BSEM^−DenseNet^, and BSEM^−VGGNet^ models, our proposed model achieved an increase of 0.8%, 1.5%, 1.1%, 0.7%, and 1.0%; 0.3%, 1.0%, 0.8%, 0.2%, and 0.5%; and 1.7%, 2.6%, 2.2%, 1.1%, and 1.4%, respectively, for accuracy, AUC, precision, recall, and F1 score, respectively (Table 2).

**Table 2:** Comparison of the model without the voting ensemble strategy and the BSEM model

Method	Accuracy	AUC	Precision	Recall	F1 score
BSEM^−ResNet^	74.3%	85.8%	75.4%	72.7%	73.1%
BSEM^−DenseNet^	74.8%	86.3%	75.7%	73.2%	73.7%
BSEM^−VGGNet^	73.4%	84.7%	74.3%	72.3%	72.7%
BSEM	75.1%	87.3%	76.5%	73.4%	74.1%

For the comparison between the BSEM model and the model without self-distillation, our findings demonstrated improvements of 1.8%, 0.7%, 1.3%, 1.9%, and 1.9% in accuracy, AUC, precision, recall, and F1 score, respectively, for our proposed model (Table 3).

**Table 3:** Comparison between the model without the self-distillation technology and the BSEM model

Method	Accuracy	AUC	Precision	Recall	F1 score
ResNet	70.7%	83.4%	72.6%	68.6%	69.3%
DenseNet	71.8%	84.1%	72.3%	70.3%	70.8%
VGGNet	70.0%	81.8%	71.5%	67.7%	68.4%
BSEM^−SD^	73.3%	86.6%	75.2%	71.5%	72.2%
BSEM	75.1%	87.3%	76.5%	73.4%	74.1%

The ablation experiments demonstrated the effectiveness of the voting ensemble and self-distillation in enhancing the model performance. The voting ensemble decreased the model variance and improved the robustness and accuracy. Meanwhile, self-distillation improved model generalisation and classification abilities by aggregating knowledge. Consequently, both voting ensembles and self-distillation were crucial and effective for enhancing the model performance, further confirming the superiority of the BSEM model presented in this paper.

## Discussion

Endometrial cancer is the sixth most commonly diagnosed cancer in women (Sung et al. 2021). Early-stage endometrial disease has a high cure rate (Makker et al. 2021), and ultrasound is the preferred method for early diagnosis (Wong et al. 2016). However, conventional classification methods for endometrial diseases typically rely on the manual examination and analysis of numerous medical images, which are time-consuming processes with subjective errors. Conversely, deep learning models possess robust feature extraction and learning capabilities, enabling the automatic extraction of crucial features from input data (Xu et al. 2022; Wei et al. 2023; Yang et al. 2022; Chen et al. 2022). This aids radiologists in determining the disease type and supports their decision-making processes, thereby enhancing diagnostic accuracy. Furthermore, deep learning models significantly improve processing speed and efficiency through their automatic classification and identification capabilities, thereby alleviating the workload burden on radiologists (Ker et al. 2017; Liu et al. 2019).

However, the use of deep learning for the classification of endometrial diseases still faces certain challenges (Zhang et al. 2022, 2021; Li et al. 2022; Urushibara et al. 2022; Mao et al. 2022; Tao et al. 2022; Zhao et al. 2022; Sun et al. 2020), such as incomplete classification, limited sample diversity, and dependence on specific data such as MRI and HI, resulting in low model robustness and generalisation. To address these concerns, this study proposed the BSEM model, which utilised TVU images as raw data for disease classification, which enhanced the model performance by training a joint classifier on the original and self-supervised tasks. In addition, we incorporated self-distillation technology to reduce the dependence on specific image types and enhance the generalisation of the model, as shown in Table 3. Moreover, we used a voting ensemble strategy to minimise the model variance and improve the overall performance and stability, as shown in Table 2.

During the model-testing phase, a fivefold cross-validation approach was used to evaluate the performance of the model. The results demonstrated that the BSEM model was advantageous for the classification of endometrial diseases, with improved accuracy, AUC, precision, recall, and F1 score; the values achieved were 75.1%, 87.3%, 76.5%, 73.4%, and 74.1%, respectively. Compared with the baseline ResNet model, our model exhibited improvements of 4.4%, 3.9%, 3.9%, 4.8%, and 4.8%, for accuracy, AUC, precision, recall, and F1 score, respectively. Furthermore, compared with the baseline DenseNet model, our model exhibited enhancements of 3.3%, 3.2%, 4.2%, 3.1%, and 3.3%, respectively. In comparison to the baseline VGGNet model, our model indicated advancements of 5.1%, 5.5%, 5.0%, 5.7%, and 5.7%, respectively. Then, compared with the baseline ConvNeXt model, our model displayed enhancements of 5.2%, 5.1%, 7.2%, 5.0%, and 5.6%, respectively. In comparison to the baseline VIT model, our model showed advancements of 6.6%, 7.0%, 7.4%, 6.7%, and 6.8%, respectively. Finally, compared with the baseline CMT model, our model demonstrated enhancements of 7.9%, 7.3%, 8.5%, 8.5%, and 9.0%, respectively. These results confirmed the viability and effectiveness of deep learning techniques for the diagnosis of endometrial diseases.

Our study had some limitations. While cropping the image, we were unable to simply keep the lesion area, which could have affected the accuracy of the model in lesion detection and detailed lesion analysis. Also, the limited amount of data on endometrial cancer compared with those for endometrial polyps and hyperplasia could potentially lead to suboptimal classification results for cancer cases. To address these limitations, future research should focus on refining image-processing techniques and exploring more precise methods for extracting lesion regions to enhance the accuracy and completeness of lesion identification in cropped images. Additionally, augmenting the size of cancer datasets to facilitate the comprehensive training and evaluation of models represents a crucial avenue for improving cancer classification performance.

## Conclusion

This study proposed the BSEM model, a model for endometrial disease classification using TVU images that combines original and self-supervised tasks and incorporates the self-distillation technique and voting ensemble strategy. Specific diseases targeted were endometrial cancer, polyps, and hyperplasia. The performance of the model was evaluated using a fivefold cross-validation method during testing, and the experimental results demonstrated its high generalisability and robustness.

## Data Availability

The datasets generated during and/or analysed during the current study are available from the corresponding author on reasonable request.
